# Synthesis and structure of push–pull merocyanines based on barbituric and thio­barbituric acid

**DOI:** 10.1107/S2056989019011071

**Published:** 2019-08-16

**Authors:** Georgii Bogdanov, John P. Tillotson, Jenna Bustos, Tatiana V. Timofeeva

**Affiliations:** a Department of Chemistry, New Mexico Highlands University, Las Vegas, New Mexico, 87701, USA; b School of Chemistry and Biochemistry, Georgia Institute of Technology, Atlanta, Georgia, 30332, USA

**Keywords:** crystal structure, barbituric acid derivatives, push–pull chromophores

## Abstract

Two compounds, 1,3-diethyl-5-{(2*E*,4*E*)-6-[(*E*)-1,3,3-tri­methyl­indolin-2-yl­idene]hexa-2,4-dien-1-yl­idene}pyrimidine-2,4,6(1*H*,3*H*,5*H*)-trione or TMI, C_25_H_29_N_3_O_3_, and 1,3-diethyl-2-sulfanyl­idene-5-[2-(1,3,3-tri­methyl­indolin-2-yl­idene)ethyl­idene]di­hydro­pyrimidine-4,6(1*H*,5*H*)-dione or DTB, C_21_H_25_N_3_O_2_S, have been crystallized and studied. These compounds contain the same indole derivative donor group and differ in their acceptor groups (in TMI it contains oxygen in the *para* position, and in DTB sulfur) and the length of the π-bridge. In both materials, mol­ecules are packed in a herringbone manner with differences in the twist and fold angles. In both structures, the mol­ecules are connected by weak C—H⋯O and/or C—H⋯S bonds.

## Abstract 2

The synthesis and crystal structures of 1,3-diethyl-5-{(2*E*,4*E*)-6-[(*E*)-1,3,3-tri­methyl­indolin-2-yl­idene]hexa-2,4-dien-1-yl­idene}pyrimidine-2,4,6(1*H*,3*H*,5*H*)-trione or TMI, C_25_H_29_N_3_O_3_, and 1,3-diethyl-2-sulfanyl­idene-5-[2-(1,3,3-tri­methyl­indolin-2-yl­idene)ethyl­idene]di­hydro­pyrimidine-4,6(1*H*,5*H*)-dione or DTB, C_21_H_25_N_3_O_2_S, are described. These compounds contain the same indole derivative donor group and differ in their acceptor groups (in TMI it contains oxygen in the *para* position, and in DTB sulfur) and the length of the π-bridge.

## Chemical context   

The structures and properties of merocyanine dyes that lead to their potential use as non-linear optical materials have been studied widely over the past several decades (Del Zoppo *et al.*, 1998 ▸; Bublitz & Boxer, 1998 ▸; Kulinich *et al.*, 2007 ▸; Liess *et al.*, 2015 ▸). For so-called push–pull systems with donor and acceptor groups connected by a π-conjugated bridge, non-linear optical applications are possible as a result of the charge-transfer phenomenon within one mol­ecule. As previously reported (Klikar *et al.*, 2013 ▸; Bideau *et al.*, 1976 ▸, 1977 ▸; Bublitz, Ortiz, Marder *et al.*, 1997 ▸; Bourhill *et al.*, 1994 ▸), studies of mol­ecules with barbituric or thio­barbituric acid as acceptor (Adamson *et al.*, 1999 ▸; Padgett *et al.*, 2007 ▸) show high values of first hyperpolarizability. Recently, more applications in the biological field have also been reported for such compounds (Collot *et al.*; 2018 ▸, Golovnev *et al.*, 2018 ▸; Molokeev *et al.*, 2015 ▸) related to their ability of bright fluorescence. Both structures reported here have the same 1,3,3-trimethyl-2-methyl­eneindoline moiety as a donor group. Studies of mol­ecules with different lengths of the π-bridge between the donor and acceptor groups (Ortiz *et al.*, 1994 ▸, Vázquez-Vuelvas *et al.*, 2011 ▸) have demonstrated their different properties. Some non-linear optical studies were made on compounds with very similar structures to those presented here (Ikeda *et al.*, 1991 ▸; Chamberlain *et al.*, 1980 ▸; Kulinich *et al.*, 2008 ▸), which vary by substitutions attached to the donor or acceptor groups (Song *et al.*, 2005 ▸; Naik *et al.*, 2017 ▸; Hirshberg *et al.*, 1955 ▸). Almost all those studies were carried out in solution. Here we report the single-crystal X-ray structural analysis of two merocyanines, 1,3-diethyl-5-{(2*E*,4*E*)-6-[(*E*)-1,3,3-tri­methyl­indolin-2-yl­idene]hexa-2,4-dien-1-yl­idene}pyrimidine-2,4,6(1*H*,3*H*,5*H*)-trione or TMI, and 1,3-diethyl-2-sulfanyl­idene-5-[2-(1,3,3-tri­methyl­indolin-2-yl­idene)ethyl­idene]di­hydro­pyrimidine-4,6(1*H*,5*H*)-dione or DTB.
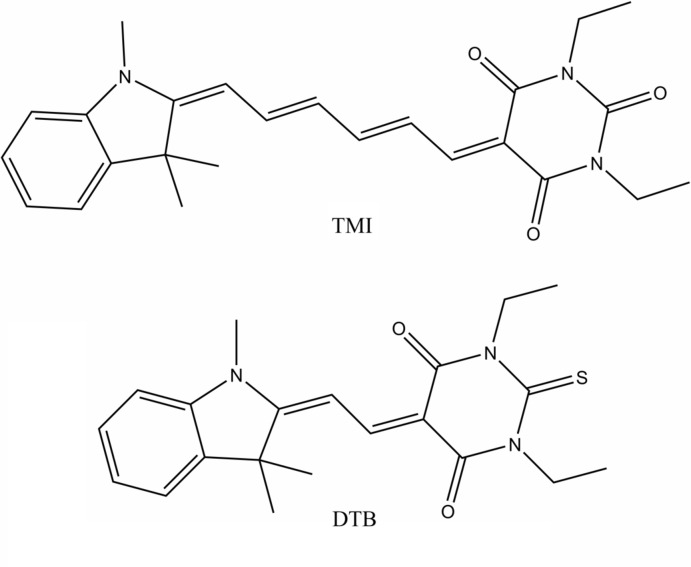


## Structural commentary   

**Figure 1:**
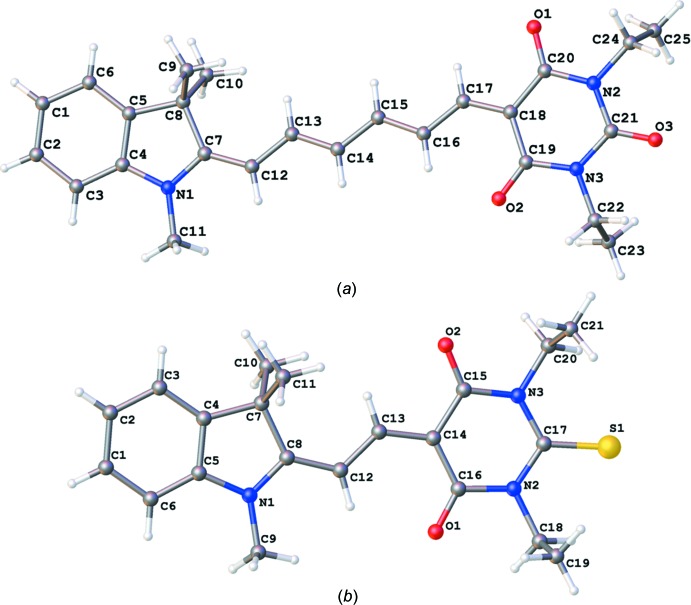
Views of the formula units of (*a*) TMI and (*b*) DTB with the atom-labelling schemes.

Both title compounds have the same donor 2,3-di­hydro-1,3,3-tri­methyl-1*H*-indole moiety with different acceptors: 1,3-di­ethyl-2-oxobarbituric acid in TMI (Fig. 1 ▸ *a*) and 1,3-di­ethyl-2-thio­barbituric acid in DTB (Fig. 1 ▸ *b*). The double and single bonds in the TMI π-bridge vary in length from 1.372 (2) to 1.410 (2) Å, the difference between the single and double bonds getting smaller closer to the acceptor, indicating a higher degree of conjugation in this region. The dihedral angles between donor group and the bridge and between the bridge and the acceptor group are 9.10 (12) and 7.44 (12)°, respectively. All three fragments of the TMI structure are slightly distorted from a planar configuration, as shown by the r.m.s. deviations of 0.022 and 0.039 Å, respectively, for atoms in the donor and acceptor groups.

Comparing DTB to TMI, it is observed that DTB possesses a more planar and rigid structure, consistent with previously reported results for studies of push–pull chromophores with different π-bridge lengths (Tillotson *et al.*, 2019 ▸). The dihedral angles between the three fragments are smaller, 3.21 (14)° between the donor group and the bridge and 1.04 (14)° between the bridge and the acceptor group. The r.m.s. deviations of atoms in DTB are also smaller, being 0.014 and 0.020 Å for the donor and acceptor groups, respectively.

In both structures, the π-bridge has an almost planar structure with insignificant r.m.s. deviations of atoms from planarity of 0.007 and 0.009 Å for TMI and DTB, respectively. In DTB, the bond-length distribution in the central fragment does not correspond to that in the scheme. According to the observed bond lengths [C8—C12 1.403 (1), C12—C13 1.386 (1), C13—C14 1.404 (1) Å], the central fragment can be presented as C8—C12=C13—C14, which indicates that the contribution of the zwitterionic form in the mol­ecular structure of DTB. It should be mentioned that measurements of the first mol­ecular hyperpolarizability, β, have positive values for dyes with hexa­methine bridges, such as TMI, while dyes with a dimethine bridge have negative β values (Ortiz *et al.*, 1994 ▸). The authors connect this effect with the high polarization and zwitterionic form of mol­ecule DTB, which has a short conjugated bridge.

## Supra­molecular features   

**Figure 2:**
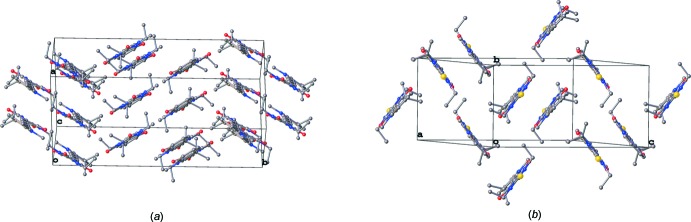
The mol­ecular packing in the crystals of compounds (*a*) TMI and (*b*) DTB.

**Figure 3:**
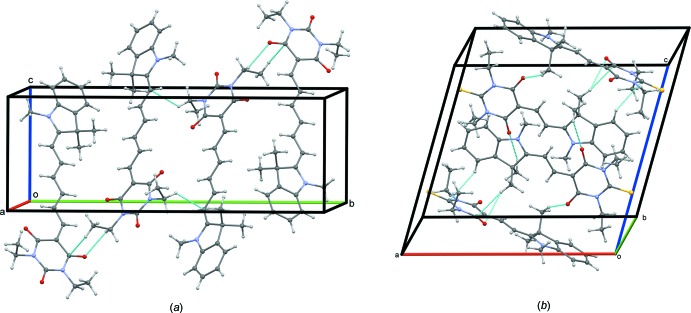
Hydrogen-bonding scheme in (*a*) TMI and (*b*) DTB.

In the crystals of both TMI and DTB mol­ecules are packed in a herringbone manner with a twist angle of 38.57 (1)° and fold angle of 57.08 (1)° in TMI (Fig. 2 ▸ *a*) and a twist angle of 54.90 (7)° and fold angle of 78.96 (3)° in DTB (Fig. 2 ▸ *b*). In both compounds, mol­ecules are hold together *via* three hydrogen bonds, of the C—H⋯O type in TMI and of the C—H⋯O and C—H⋯S types in DTB (Tables 1 ▸ and 2 ▸, Fig. 3 ▸).

For push–pull mol­ecules be applied in the form of non-linear crystalline materials, they should exhibit a non-centrosymmetric type of packing. TMI and DTB both crystallize in the centrosymmetric space group *P*2_1_/*c*. According to the bond order alternation pattern in these structures (see the supporting information), we suggest that they have the potential to be used as non-linear optical materials, but for this application they should be either be embedded in a polymer matrix or recrystallized under different conditions to attain an acentric packing mode.

## Database survey   

**Figure 4:**
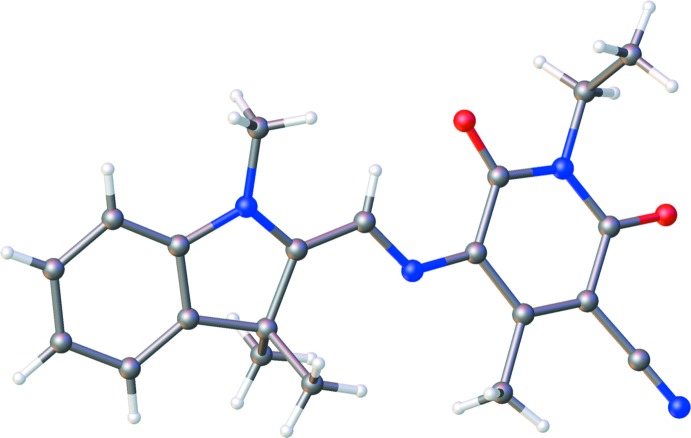
The mol­eculular structure of a compound with a similar structure to DTB (PAQYEM; Song *et al.*, 2005 ▸).

**Figure 5:**
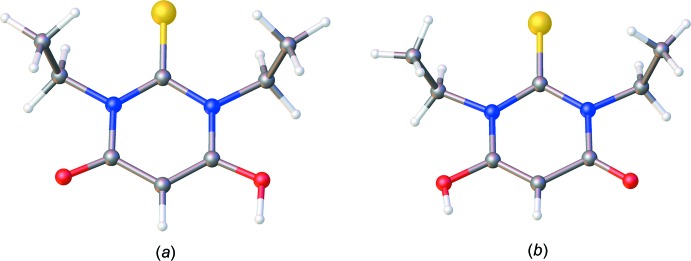
The mol­ecular structures of compounds with an acceptor group very close to those in the chromophores reported here: (*a*) DETSBR01 (Bideau *et al.*, 1977 ▸) and (*b*) DETSBR11 (Padgett *et al.*, 2007 ▸).

The Cambridge Structural Database (CSD version 5.40, last update November 2018; Groom *et al.*, 2016 ▸) was searched three times: for the donor group, which is the same for both studied structures, and separately for each acceptor group. A search for the full structures gave zero hits. The dependence of the first hyperpolarizability on polarization and the length of the π-bridge that comprises donor or acceptors of studied mol­ecules is described in several publications [KIYTOC and KOFMAU, Kulinich *et al.*, 2007 ▸; GUBDAK, Liess *et al.*, 2015 ▸ (Fig. 4 ▸); POLZEV, Ortiz *et al.*, 1994 ▸; WIMHAD and WIMHEH, Klikar *et al.*, 2013 ▸; WEVMUF, Bourhill *et al.*, 1994 ▸]. In addition, the acceptor group of the TMI structure has been studied separately and the results were published (DETBAR10; Bideau *et al.*, 1976 ▸). The acceptor group of DTB was studied as an independent mol­ecule (DETSBR10; Bideau *et al.*, 1976 ▸), as a part of several chromophore mol­ecules (GUDWEH, Adamson *et al.*, 1999 ▸; GUDWEH01, Naik *et al.*, 2017 ▸; WEVMUF, Bourhill *et al.*, 1994 ▸) and also as an anion in complexes with different cations (HUKMAD, HUKMEH, HUKMIL and HUKMOR; Molokeev *et al.*, 2015 ▸). We found several publications in which the mol­ecules are similar to our donor and acceptors, for instance PAQYEM (Song *et al.*, 2005 ▸) is similar to TMI, but with a methyl group instead of an oxygen atom in the *ortho* position of the acceptor ring, and a cyano group in the *meta* position instead of an ethyl group (Fig. 4 ▸). Two structures of the separately crystallized acceptor group (DETSBR01, Bideau *et al.*, 1977 ▸; DETSBR11, Padgett *et al.*, 2007 ▸) are very close to that of the acceptor of DTB, but with hy­droxy groups in the *ortho* positions instead of carbonyl oxygen atoms (Fig. 5 ▸).

## Synthesis and crystallization   

**Figure 6:**
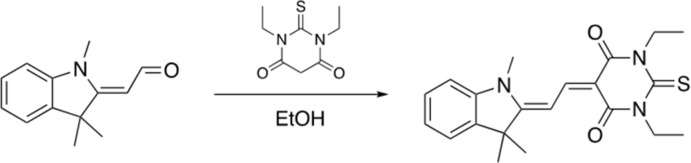
Synthesis of 1,3-diethyl-2-thioxo-5-(2-(1,3,3-tri­methyl­indolin-2-yl­idene)ethyl­idene)di­hydro­pyrimidine-4,6(1*H*,5*H*)-dione (DTB).

The synthesis of TMI was described by Ortiz *et al.* (1994 ▸), and this material was kindly presented to our group for structural studies by Professor Marderr’s group. A scheme for the synthesis of DTB is shown in Fig. 6 ▸.

*Synthesis of 1,3-diethyl-2-sulfanyl­idene-5-[2-(1,3,3-tri­meth­yl­indolin-2-yl­idene)ethyl­idene]di­hydro­pyrimidine-4,6(1H,5H)-dione (DTB)*:

2-(1,3,3-Tri­methyl­indolin-2-yl­idene)acetaldehyde (0.25 g, 1.2 mmol) and diethyl­thio­barbituric acid (0.25 g, 1.24 mmol) were dissolved in about 35 mL of absolute ethanol with stirring and sonication. After stirring for 1 h at room temperature, the product was precipitated by adding distilled water. The mixture was then filtered and the residue redissolved in EtOH and precipitated again. The precipitant was washed with hexane and dried *in vacuo* to give 1,3-diethyl-2-sulfanyl­idene-5-[2-(1,3,3-tri­methyl­indolin-2-yl­idene)ethyl­idene]di­hydro­pyrimidine-4,6(1*H*,5*H*)-dione as transparent red crystals (0.41 g, 86% yield). ^1^H NMR 8.69 (*d*, *J* = 14.6 Hz, 1H), 7.70 (*d*, *J* = 14.6 Hz, 1H), 7.40 (*m*, 2H), 7.26 (*t*, *J* = 7.9 Hz, 1H), 7.12 (*d*, *J* = 8.6 Hz, 1H), 4.55 (*q*, *J* = 7.0 Hz, 2H), 4.54 (*q*, *J* = 7.0 Hz, 2H), 3.59 (*s*, 3H), 1.73 (*s*, 6H), 1.27 (*t*, *J* = 7.0 Hz, 3H), 1.26 (*t*, *J* = 7.0 Hz, 3H) ppm.

Single crystals of both DTB and TMI were grown by vapour diffusion using chloro­form as the solvent and cyclo­hexane as the anti­solvent. Crystallization took place over a three week period to give DTB crystals of suitable size and quality.

## Refinement   

Crystal data, data collection and structure refinement details are summarized in Table 3 ▸. The hydrogen atoms on the aromatic ring of the donor group and the π-bridge in both structures were positioned geometrically, C—H = 0.95 Å. Other hydrogens were positioned with idealized geometries C—H = 0.98–0.99 Å. All H atoms were refined using a riding model with *U*_iso_(H) = 1.2*U*_eq_(C) or 1.5*U*_eq_(C-meth­yl).

## Supplementary Material

Crystal structure: contains datablock(s) TMI, DTB. DOI: 10.1107/S2056989019011071/yk2125sup1.cif

Structure factors: contains datablock(s) TMI. DOI: 10.1107/S2056989019011071/yk2125TMIsup2.hkl

Structure factors: contains datablock(s) DTB. DOI: 10.1107/S2056989019011071/yk2125DTBsup3.hkl

Supporting information file. DOI: 10.1107/S2056989019011071/yk2125TMIsup4.cml

Supporting information file. DOI: 10.1107/S2056989019011071/yk2125DTBsup5.cml

CCDC references: 1946233, 1946232

Additional supporting information:  crystallographic information; 3D view; checkCIF report

## Figures and Tables

**Table 1:** Hydrogen-bond geometry (Å, °) for TMI[Chem scheme1]

*D*—H⋯*A*	*D*—H	H⋯*A*	*D*⋯*A*	*D*—H⋯*A*
C3—H3⋯O2^i^	0.95	2.61	3.5458 (17)	167
C24—H24*B*⋯O1^ii^	0.99	2.56	3.2939 (16)	131
C11—H11*A*⋯O2^i^	0.98	2.43	3.3891 (17)	165

Symmetry codes: (i) 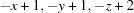; (ii) 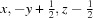.

**Table 2:** Hydrogen-bond geometry (Å, °) for DTB[Chem scheme1]

*D*—H⋯*A*	*D*—H	H⋯*A*	*D*⋯*A*	*D*—H⋯*A*
C12—H12⋯O1	0.95	2.28	2.9000 (10)	122
C19—H19*B*⋯S1^i^	0.98	2.85	3.5573 (9)	130
C21—H21*A*⋯S1	0.98	2.98	3.4965 (12)	114

Symmetry code: (i) 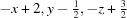.

**Table 3:** Experimental details

	TMI	DTB
Crystal data		
Chemical formula	C_25_H_29_N_3_O_3_	C_21_H_25_N_3_O_2_S
*M*_r_	419.51	383.50
Crystal system, space group	Monoclinic, *P*2_1_/*c*	Monoclinic, *P*2_1_/*c*
Temperature (K)	100	100
*a*, *b*, *c* (Å)	11.7624 (9), 22.9546 (19), 8.1934 (7)	16.1504 (6), 8.1264 (3), 15.6487 (6)
β (°)	93.717 (2)	108.849 (1)
*V* (Å^3^)	2207.6 (3)	1943.67 (13)
*Z*	4	4
Radiation type	Mo *K*α	Mo *K*α
μ (mm^−1^)	0.08	0.19
Crystal size (mm)	0.3 × 0.25 × 0.11	0.3 × 0.26 × 0.24

Data collection		
Diffractometer	Bruker APEXII CCD	Bruker APEXII CCD
Absorption correction	Multi-scan (*SADABS*; Bruker, 2016 ▸)	Multi-scan (*SADABS*; Bruker, 2016 ▸)
*T*_min_, *T*_max_	0.601, 0.746	0.678, 0.748
No. of measured, independent and observed [*I* > 2σ(*I*)] reflections	69450, 7037, 4810	92854, 12354, 8903
*R*_int_	0.085	0.045
(sin θ/λ)_max_ (Å^−1^)	0.726	0.913

Refinement		
*R*[*F* ^2^ > 2σ(*F* ^2^)], *wR*(*F* ^2^), *S*	0.048, 0.117, 1.02	0.046, 0.141, 1.04
No. of reflections	7037	12354
No. of parameters	285	249
H-atom treatment	H-atom parameters constrained	H-atom parameters constrained
Δρ_max_, Δρ_min_ (e Å^−3^)	0.33, −0.28	0.64, −0.46

Computer programs: *APEX3* and *SAINT* (Bruker, 2016 ▸), *SHELXT* (Sheldrick, 2015*a* ▸), *SHELXL* (Sheldrick, 2015*b* ▸) and *OLEX2* (Dolomanov *et al.*, 2009 ▸).

## supplementary crystallographic information

### 1,3-Diethyl-5-{(2E,4E)-6-[(E)-1,3,3-trimethylindolin-2-ylidene]hexa-2,4-dien-1-ylidene}pyrimidine-2,4,6(1H,3H,5H)-trione (TMI) Crystal data

**Table:**

C_25_H_29_N_3_O_3_	*F*(000) = 896
*M**_r_* = 419.51	*D*_x_ = 1.262 Mg m^−^^3^
Monoclinic, *P*2_1_/*c*	Mo *K*α radiation, λ = 0.71073 Å
*a* = 11.7624 (9) Å	Cell parameters from 8868 reflections
*b* = 22.9546 (19) Å	θ = 2.5–28.2°
*c* = 8.1934 (7) Å	µ = 0.08 mm^−^^1^
β = 93.717 (2)°	*T* = 100 K
*V* = 2207.6 (3) Å^3^	Plate, metallic light blue
*Z* = 4	0.3 × 0.25 × 0.11 mm

### 1,3-Diethyl-5-{(2E,4E)-6-[(E)-1,3,3-trimethylindolin-2-ylidene]hexa-2,4-dien-1-ylidene}pyrimidine-2,4,6(1H,3H,5H)-trione (TMI) Data collection

**Table:**

Bruker APEXII CCD diffractometer	4810 reflections with *I* > 2σ(*I*)
φ and ω scans	*R*_int_ = 0.085
Absorption correction: multi-scan (SADABS; Bruker, 2016)	θ_max_ = 31.1°, θ_min_ = 1.7°
*T*_min_ = 0.601, *T*_max_ = 0.746	*h* = −16→17
69450 measured reflections	*k* = −33→33
7037 independent reflections	*l* = −11→11

### 1,3-Diethyl-5-{(2E,4E)-6-[(E)-1,3,3-trimethylindolin-2-ylidene]hexa-2,4-dien-1-ylidene}pyrimidine-2,4,6(1H,3H,5H)-trione (TMI) Refinement

**Table:**

Refinement on *F*^2^	Primary atom site location: dual
Least-squares matrix: full	Secondary atom site location: difference Fourier map
*R*[*F*^2^ > 2σ(*F*^2^)] = 0.048	Hydrogen site location: inferred from neighbouring sites
*wR*(*F*^2^) = 0.117	H-atom parameters constrained
*S* = 1.02	*w* = 1/[σ^2^(*F*_o_^2^) + (0.0433*P*)^2^ + 0.7306*P*] where *P* = (*F*_o_^2^ + 2*F*_c_^2^)/3
7037 reflections	(Δ/σ)_max_ < 0.001
285 parameters	Δρ_max_ = 0.33 e Å^−^^3^
0 restraints	Δρ_min_ = −0.28 e Å^−^^3^

### 1,3-Diethyl-5-{(2E,4E)-6-[(E)-1,3,3-trimethylindolin-2-ylidene]hexa-2,4-dien-1-ylidene}pyrimidine-2,4,6(1H,3H,5H)-trione (TMI) Special details

**Table:**

Geometry. All esds (except the esd in the dihedral angle between two l.s. planes) are estimated using the full covariance matrix. The cell esds are taken into account individually in the estimation of esds in distances, angles and torsion angles; correlations between esds in cell parameters are only used when they are defined by crystal symmetry. An approximate (isotropic) treatment of cell esds is used for estimating esds involving l.s. planes.

### 1,3-Diethyl-5-{(2E,4E)-6-[(E)-1,3,3-trimethylindolin-2-ylidene]hexa-2,4-dien-1-ylidene}pyrimidine-2,4,6(1H,3H,5H)-trione (TMI) Fractional atomic coordinates and isotropic or equivalent isotropic displacement parameters (Å^2^)

**Table:**

	*x*	*y*	*z*	*U*_iso_*/*U*_eq_	
O1	0.57141 (8)	0.28544 (4)	0.06369 (11)	0.0250 (2)	
C14	0.45141 (11)	0.39420 (5)	0.74941 (16)	0.0210 (3)	
H14	0.486034	0.431317	0.765679	0.025*	
O3	0.84399 (9)	0.40063 (4)	−0.13659 (12)	0.0300 (2)	
O2	0.68018 (9)	0.46420 (4)	0.31743 (12)	0.0314 (2)	
N1	0.28095 (9)	0.42110 (4)	1.26866 (12)	0.0195 (2)	
N2	0.70066 (9)	0.34541 (5)	−0.04349 (13)	0.0203 (2)	
N3	0.75975 (9)	0.43289 (4)	0.08893 (13)	0.0201 (2)	
C7	0.29334 (10)	0.38885 (5)	1.13030 (15)	0.0172 (2)	
C8	0.21355 (10)	0.33601 (5)	1.13327 (15)	0.0174 (2)	
C5	0.15411 (11)	0.34692 (5)	1.28882 (15)	0.0187 (2)	
C12	0.36413 (11)	0.40500 (5)	1.01155 (15)	0.0196 (2)	
H12	0.404050	0.440701	1.028219	0.023*	
C4	0.19733 (11)	0.39734 (5)	1.36456 (15)	0.0195 (2)	
C13	0.38377 (11)	0.37410 (5)	0.86730 (15)	0.0201 (2)	
H13	0.348086	0.337243	0.850912	0.024*	
C20	0.62694 (11)	0.33057 (5)	0.07785 (15)	0.0195 (2)	
C17	0.55933 (10)	0.35433 (5)	0.34446 (15)	0.0201 (2)	
H17	0.523420	0.317338	0.333554	0.024*	
C21	0.77321 (11)	0.39312 (6)	−0.03588 (15)	0.0212 (3)	
C15	0.47303 (11)	0.36355 (6)	0.60582 (16)	0.0209 (3)	
H15	0.439083	0.326297	0.589055	0.025*	
C18	0.62303 (10)	0.37085 (5)	0.21547 (15)	0.0188 (2)	
C19	0.68610 (11)	0.42537 (5)	0.21503 (15)	0.0206 (3)	
C16	0.54072 (11)	0.38470 (6)	0.48855 (16)	0.0214 (3)	
H16	0.576385	0.421508	0.506047	0.026*	
C22	0.83708 (11)	0.48360 (5)	0.09856 (16)	0.0225 (3)	
H22A	0.797739	0.517098	0.146023	0.027*	
H22B	0.856797	0.494552	−0.013005	0.027*	
C9	0.12617 (11)	0.33600 (6)	0.98519 (15)	0.0218 (3)	
H9A	0.069422	0.305443	0.999149	0.033*	
H9B	0.165282	0.328573	0.885221	0.033*	
H9C	0.088140	0.373965	0.976858	0.033*	
C6	0.06730 (11)	0.31656 (6)	1.35644 (16)	0.0229 (3)	
H6	0.037084	0.282200	1.305626	0.028*	
C3	0.15744 (12)	0.41847 (6)	1.50894 (16)	0.0243 (3)	
H3	0.188713	0.452496	1.560313	0.029*	
C10	0.28018 (11)	0.27818 (5)	1.14518 (17)	0.0230 (3)	
H10A	0.226542	0.245599	1.148562	0.034*	
H10B	0.330873	0.278119	1.244935	0.034*	
H10C	0.325495	0.274050	1.049600	0.034*	
C24	0.70962 (12)	0.30531 (6)	−0.18308 (15)	0.0235 (3)	
H24A	0.730561	0.327686	−0.279980	0.028*	
H24B	0.634697	0.286813	−0.209861	0.028*	
C2	0.06930 (12)	0.38748 (6)	1.57531 (16)	0.0267 (3)	
H2	0.039178	0.400960	1.673280	0.032*	
C1	0.02468 (12)	0.33723 (6)	1.50078 (17)	0.0272 (3)	
H1	−0.035165	0.316835	1.548404	0.033*	
C11	0.34104 (12)	0.47513 (6)	1.30867 (17)	0.0255 (3)	
H11A	0.331476	0.485237	1.423182	0.038*	
H11B	0.309903	0.506406	1.237660	0.038*	
H11C	0.422246	0.470163	1.292140	0.038*	
C25	0.79828 (12)	0.25841 (6)	−0.14451 (18)	0.0289 (3)	
H25A	0.805058	0.233709	−0.240946	0.043*	
H25B	0.775041	0.234562	−0.053227	0.043*	
H25C	0.871977	0.276688	−0.114467	0.043*	
C23	0.94519 (12)	0.47040 (6)	0.20246 (19)	0.0297 (3)	
H23A	0.994861	0.504712	0.206046	0.045*	
H23B	0.984717	0.437534	0.154931	0.045*	
H23C	0.925955	0.460408	0.313647	0.045*	

### 1,3-Diethyl-5-{(2E,4E)-6-[(E)-1,3,3-trimethylindolin-2-ylidene]hexa-2,4-dien-1-ylidene}pyrimidine-2,4,6(1H,3H,5H)-trione (TMI) Atomic displacement parameters (Å^2^)

**Table:**

	*U*^11^	*U*^22^	*U*^33^	*U*^12^	*U*^13^	*U*^23^
O1	0.0246 (5)	0.0215 (5)	0.0289 (5)	−0.0026 (4)	0.0012 (4)	−0.0021 (4)
C14	0.0197 (6)	0.0193 (6)	0.0243 (6)	−0.0012 (5)	0.0032 (5)	0.0022 (5)
O3	0.0326 (6)	0.0326 (5)	0.0261 (5)	−0.0046 (4)	0.0110 (4)	−0.0023 (4)
O2	0.0397 (6)	0.0235 (5)	0.0325 (5)	−0.0086 (4)	0.0153 (5)	−0.0079 (4)
N1	0.0213 (5)	0.0172 (5)	0.0200 (5)	−0.0035 (4)	0.0015 (4)	−0.0014 (4)
N2	0.0204 (5)	0.0212 (5)	0.0193 (5)	0.0011 (4)	0.0011 (4)	−0.0016 (4)
N3	0.0208 (5)	0.0184 (5)	0.0214 (5)	−0.0011 (4)	0.0047 (4)	0.0003 (4)
C7	0.0171 (6)	0.0148 (5)	0.0193 (5)	0.0003 (4)	−0.0010 (4)	0.0008 (4)
C8	0.0171 (6)	0.0151 (5)	0.0200 (5)	−0.0011 (4)	0.0018 (4)	−0.0005 (4)
C5	0.0190 (6)	0.0182 (5)	0.0186 (5)	0.0007 (5)	−0.0001 (4)	0.0017 (4)
C12	0.0184 (6)	0.0169 (5)	0.0234 (6)	−0.0015 (5)	0.0014 (5)	0.0013 (5)
C4	0.0197 (6)	0.0202 (6)	0.0183 (5)	−0.0003 (5)	−0.0006 (5)	0.0019 (4)
C13	0.0170 (6)	0.0184 (6)	0.0248 (6)	0.0005 (5)	0.0014 (5)	0.0023 (5)
C20	0.0170 (6)	0.0204 (6)	0.0210 (6)	0.0037 (5)	0.0001 (5)	0.0014 (5)
C17	0.0155 (6)	0.0197 (6)	0.0250 (6)	0.0000 (5)	0.0009 (5)	0.0006 (5)
C21	0.0210 (6)	0.0222 (6)	0.0203 (6)	0.0019 (5)	0.0012 (5)	0.0006 (5)
C15	0.0174 (6)	0.0199 (6)	0.0256 (6)	−0.0002 (5)	0.0025 (5)	0.0021 (5)
C18	0.0167 (6)	0.0185 (6)	0.0211 (6)	0.0008 (4)	0.0011 (4)	0.0001 (5)
C19	0.0202 (6)	0.0202 (6)	0.0217 (6)	0.0014 (5)	0.0033 (5)	0.0009 (5)
C16	0.0181 (6)	0.0212 (6)	0.0250 (6)	−0.0006 (5)	0.0024 (5)	0.0011 (5)
C22	0.0247 (7)	0.0182 (6)	0.0253 (6)	−0.0026 (5)	0.0060 (5)	0.0020 (5)
C9	0.0205 (6)	0.0243 (6)	0.0204 (6)	−0.0008 (5)	−0.0006 (5)	−0.0031 (5)
C6	0.0234 (7)	0.0216 (6)	0.0239 (6)	−0.0028 (5)	0.0025 (5)	0.0013 (5)
C3	0.0287 (7)	0.0246 (6)	0.0194 (6)	−0.0008 (5)	−0.0007 (5)	−0.0015 (5)
C10	0.0223 (6)	0.0166 (6)	0.0302 (7)	0.0004 (5)	0.0036 (5)	0.0015 (5)
C24	0.0254 (7)	0.0263 (6)	0.0187 (6)	0.0022 (5)	−0.0001 (5)	−0.0041 (5)
C2	0.0313 (7)	0.0303 (7)	0.0190 (6)	0.0013 (6)	0.0044 (5)	0.0014 (5)
C1	0.0269 (7)	0.0307 (7)	0.0245 (6)	−0.0037 (6)	0.0060 (5)	0.0041 (5)
C11	0.0294 (7)	0.0184 (6)	0.0283 (7)	−0.0063 (5)	−0.0013 (6)	−0.0032 (5)
C25	0.0254 (7)	0.0301 (7)	0.0308 (7)	0.0060 (6)	−0.0025 (6)	−0.0096 (6)
C23	0.0219 (7)	0.0270 (7)	0.0402 (8)	−0.0001 (5)	0.0020 (6)	−0.0045 (6)

### 1,3-Diethyl-5-{(2E,4E)-6-[(E)-1,3,3-trimethylindolin-2-ylidene]hexa-2,4-dien-1-ylidene}pyrimidine-2,4,6(1H,3H,5H)-trione (TMI) Geometric parameters (Å, º)

**Table:**

O1—C20	1.2261 (15)	C15—C16	1.3756 (18)
C14—H14	0.9500	C18—C19	1.4550 (17)
C14—C13	1.3706 (18)	C16—H16	0.9500
C14—C15	1.4080 (18)	C22—H22A	0.9900
O3—C21	1.2218 (16)	C22—H22B	0.9900
O2—C19	1.2291 (15)	C22—C23	1.5144 (19)
N1—C7	1.3696 (15)	C9—H9A	0.9800
N1—C4	1.4080 (16)	C9—H9B	0.9800
N1—C11	1.4544 (16)	C9—H9C	0.9800
N2—C20	1.4029 (16)	C6—H6	0.9500
N2—C21	1.3873 (16)	C6—C1	1.3971 (19)
N2—C24	1.4772 (16)	C3—H3	0.9500
N3—C21	1.3876 (16)	C3—C2	1.3964 (19)
N3—C19	1.4014 (16)	C10—H10A	0.9800
N3—C22	1.4760 (16)	C10—H10B	0.9800
C7—C8	1.5347 (16)	C10—H10C	0.9800
C7—C12	1.3721 (17)	C24—H24A	0.9900
C8—C5	1.5137 (17)	C24—H24B	0.9900
C8—C9	1.5385 (17)	C24—C25	1.5177 (19)
C8—C10	1.5414 (17)	C2—H2	0.9500
C5—C4	1.3936 (17)	C2—C1	1.392 (2)
C5—C6	1.3816 (18)	C1—H1	0.9500
C12—H12	0.9500	C11—H11A	0.9800
C12—C13	1.4102 (18)	C11—H11B	0.9800
C4—C3	1.3881 (18)	C11—H11C	0.9800
C13—H13	0.9500	C25—H25A	0.9800
C20—C18	1.4614 (17)	C25—H25B	0.9800
C17—H17	0.9500	C25—H25C	0.9800
C17—C18	1.3874 (17)	C23—H23A	0.9800
C17—C16	1.4004 (18)	C23—H23B	0.9800
C15—H15	0.9500	C23—H23C	0.9800
			
C13—C14—H14	117.8	N3—C22—H22A	109.4
C13—C14—C15	124.41 (12)	N3—C22—H22B	109.4
C15—C14—H14	117.8	N3—C22—C23	111.37 (11)
C7—N1—C4	111.60 (10)	H22A—C22—H22B	108.0
C7—N1—C11	124.84 (11)	C23—C22—H22A	109.4
C4—N1—C11	123.46 (11)	C23—C22—H22B	109.4
C20—N2—C24	118.41 (10)	C8—C9—H9A	109.5
C21—N2—C20	124.51 (11)	C8—C9—H9B	109.5
C21—N2—C24	116.88 (11)	C8—C9—H9C	109.5
C21—N3—C19	124.60 (11)	H9A—C9—H9B	109.5
C21—N3—C22	117.31 (10)	H9A—C9—H9C	109.5
C19—N3—C22	117.77 (10)	H9B—C9—H9C	109.5
N1—C7—C8	108.43 (10)	C5—C6—H6	120.6
N1—C7—C12	122.88 (11)	C5—C6—C1	118.90 (12)
C12—C7—C8	128.65 (11)	C1—C6—H6	120.6
C7—C8—C9	111.44 (10)	C4—C3—H3	121.5
C7—C8—C10	111.88 (10)	C4—C3—C2	117.03 (12)
C5—C8—C7	101.47 (9)	C2—C3—H3	121.5
C5—C8—C9	110.04 (10)	C8—C10—H10A	109.5
C5—C8—C10	110.48 (10)	C8—C10—H10B	109.5
C9—C8—C10	111.14 (10)	C8—C10—H10C	109.5
C4—C5—C8	109.71 (11)	H10A—C10—H10B	109.5
C6—C5—C8	130.38 (11)	H10A—C10—H10C	109.5
C6—C5—C4	119.88 (12)	H10B—C10—H10C	109.5
C7—C12—H12	116.6	N2—C24—H24A	109.4
C7—C12—C13	126.75 (12)	N2—C24—H24B	109.4
C13—C12—H12	116.6	N2—C24—C25	111.31 (10)
C5—C4—N1	108.73 (11)	H24A—C24—H24B	108.0
C3—C4—N1	128.85 (12)	C25—C24—H24A	109.4
C3—C4—C5	122.41 (12)	C25—C24—H24B	109.4
C14—C13—C12	123.73 (12)	C3—C2—H2	119.3
C14—C13—H13	118.1	C1—C2—C3	121.31 (13)
C12—C13—H13	118.1	C1—C2—H2	119.3
O1—C20—N2	119.21 (11)	C6—C1—H1	119.8
O1—C20—C18	124.36 (12)	C2—C1—C6	120.47 (13)
N2—C20—C18	116.42 (11)	C2—C1—H1	119.8
C18—C17—H17	115.6	N1—C11—H11A	109.5
C18—C17—C16	128.88 (12)	N1—C11—H11B	109.5
C16—C17—H17	115.6	N1—C11—H11C	109.5
O3—C21—N2	121.68 (12)	H11A—C11—H11B	109.5
O3—C21—N3	121.35 (12)	H11A—C11—H11C	109.5
N2—C21—N3	116.95 (11)	H11B—C11—H11C	109.5
C14—C15—H15	118.3	C24—C25—H25A	109.5
C16—C15—C14	123.46 (12)	C24—C25—H25B	109.5
C16—C15—H15	118.3	C24—C25—H25C	109.5
C17—C18—C20	117.41 (11)	H25A—C25—H25B	109.5
C17—C18—C19	122.52 (11)	H25A—C25—H25C	109.5
C19—C18—C20	120.05 (11)	H25B—C25—H25C	109.5
O2—C19—N3	118.62 (12)	C22—C23—H23A	109.5
O2—C19—C18	124.79 (12)	C22—C23—H23B	109.5
N3—C19—C18	116.58 (11)	C22—C23—H23C	109.5
C17—C16—H16	118.6	H23A—C23—H23B	109.5
C15—C16—C17	122.84 (12)	H23A—C23—H23C	109.5
C15—C16—H16	118.6	H23B—C23—H23C	109.5

### 1,3-Diethyl-5-{(2E,4E)-6-[(E)-1,3,3-trimethylindolin-2-ylidene]hexa-2,4-dien-1-ylidene}pyrimidine-2,4,6(1H,3H,5H)-trione (TMI) Hydrogen-bond geometry (Å, º)

**Table:**

*D*—H···*A*	*D*—H	H···*A*	*D*···*A*	*D*—H···*A*
C3—H3···O2^i^	0.95	2.61	3.5458 (17)	167
C24—H24*B*···O1^ii^	0.99	2.56	3.2939 (16)	131
C11—H11*A*···O2^i^	0.98	2.43	3.3891 (17)	165

Symmetry codes: (i) −*x*+1, −*y*+1, −*z*+2; (ii) *x*, −*y*+1/2, *z*−1/2.

### 1,3-Diethyl-2-sulfanylidene-5-[2-(1,3,3-trimethylindolin-2-ylidene)ethylidene]dihydropyrimidine-4,6(1H,5H)-dione (DTB) Crystal data

**Table:**

C_21_H_25_N_3_O_2_S	*F*(000) = 816
*M**_r_* = 383.50	*D*_x_ = 1.311 Mg m^−^^3^
Monoclinic, *P*2_1_/*c*	Mo *K*α radiation, λ = 0.71073 Å
*a* = 16.1504 (6) Å	Cell parameters from 9983 reflections
*b* = 8.1264 (3) Å	θ = 2.6–37.3°
*c* = 15.6487 (6) Å	µ = 0.19 mm^−^^1^
β = 108.849 (1)°	*T* = 100 K
*V* = 1943.67 (13) Å^3^	Block, clear light red
*Z* = 4	0.3 × 0.26 × 0.24 mm

### 1,3-Diethyl-2-sulfanylidene-5-[2-(1,3,3-trimethylindolin-2-ylidene)ethylidene]dihydropyrimidine-4,6(1H,5H)-dione (DTB) Data collection

**Table:**

Bruker APEXII CCD diffractometer	8903 reflections with *I* > 2σ(*I*)
φ and ω scans	*R*_int_ = 0.045
Absorption correction: multi-scan (SADABS; Bruker, 2016)	θ_max_ = 40.4°, θ_min_ = 1.3°
*T*_min_ = 0.678, *T*_max_ = 0.748	*h* = −29→29
92854 measured reflections	*k* = −14→14
12354 independent reflections	*l* = −28→28

### 1,3-Diethyl-2-sulfanylidene-5-[2-(1,3,3-trimethylindolin-2-ylidene)ethylidene]dihydropyrimidine-4,6(1H,5H)-dione (DTB) Refinement

**Table:**

Refinement on *F*^2^	Primary atom site location: dual
Least-squares matrix: full	Secondary atom site location: difference Fourier map
*R*[*F*^2^ > 2σ(*F*^2^)] = 0.046	Hydrogen site location: inferred from neighbouring sites
*wR*(*F*^2^) = 0.141	H-atom parameters constrained
*S* = 1.04	*w* = 1/[σ^2^(*F*_o_^2^) + (0.0645*P*)^2^ + 0.5305*P*] where *P* = (*F*_o_^2^ + 2*F*_c_^2^)/3
12354 reflections	(Δ/σ)_max_ = 0.002
249 parameters	Δρ_max_ = 0.64 e Å^−^^3^
0 restraints	Δρ_min_ = −0.46 e Å^−^^3^

### 1,3-Diethyl-2-sulfanylidene-5-[2-(1,3,3-trimethylindolin-2-ylidene)ethylidene]dihydropyrimidine-4,6(1H,5H)-dione (DTB) Special details

**Table:**

Geometry. All esds (except the esd in the dihedral angle between two l.s. planes) are estimated using the full covariance matrix. The cell esds are taken into account individually in the estimation of esds in distances, angles and torsion angles; correlations between esds in cell parameters are only used when they are defined by crystal symmetry. An approximate (isotropic) treatment of cell esds is used for estimating esds involving l.s. planes.

### 1,3-Diethyl-2-sulfanylidene-5-[2-(1,3,3-trimethylindolin-2-ylidene)ethylidene]dihydropyrimidine-4,6(1H,5H)-dione (DTB) Fractional atomic coordinates and isotropic or equivalent isotropic displacement parameters (Å^2^)

**Table:**

	*x*	*y*	*z*	*U*_iso_*/*U*_eq_	
S1	0.96141 (2)	0.35712 (4)	0.80259 (2)	0.03286 (7)	
O1	0.66676 (4)	0.20400 (9)	0.60288 (4)	0.02461 (12)	
N1	0.35986 (4)	0.35270 (9)	0.51788 (4)	0.01788 (11)	
O2	0.69412 (5)	0.64835 (10)	0.80519 (6)	0.0389 (2)	
N2	0.80016 (4)	0.28754 (10)	0.69633 (4)	0.02129 (12)	
N3	0.81446 (5)	0.51652 (10)	0.79244 (5)	0.02353 (13)	
C7	0.38323 (5)	0.56295 (9)	0.62627 (5)	0.01636 (11)	
C5	0.27705 (5)	0.42249 (10)	0.50767 (5)	0.01701 (11)	
C8	0.42390 (5)	0.42923 (10)	0.58292 (5)	0.01659 (11)	
C4	0.28730 (5)	0.54688 (9)	0.57129 (5)	0.01715 (11)	
C12	0.51201 (5)	0.38347 (10)	0.60429 (5)	0.01905 (12)	
H12	0.526462	0.294068	0.572630	0.023*	
C14	0.66935 (5)	0.43462 (10)	0.69647 (5)	0.01941 (12)	
C13	0.57882 (5)	0.46339 (10)	0.66971 (5)	0.01924 (12)	
H13	0.560884	0.549633	0.700776	0.023*	
C16	0.70815 (5)	0.30405 (11)	0.66025 (5)	0.01949 (13)	
C10	0.41564 (5)	0.73819 (10)	0.61661 (5)	0.01950 (12)	
H10A	0.477565	0.747867	0.652692	0.029*	
H10B	0.381410	0.818280	0.637982	0.029*	
H10C	0.408539	0.759843	0.553031	0.029*	
C3	0.21469 (5)	0.63327 (11)	0.57521 (6)	0.02240 (14)	
H3	0.220551	0.718222	0.618475	0.027*	
C6	0.19664 (5)	0.38216 (11)	0.44549 (5)	0.02134 (13)	
H6	0.191296	0.298193	0.401796	0.026*	
C11	0.39728 (5)	0.52071 (11)	0.72630 (5)	0.02033 (13)	
H11A	0.377983	0.407614	0.730697	0.030*	
H11B	0.363251	0.596715	0.750444	0.030*	
H11C	0.459504	0.531035	0.761164	0.030*	
C1	0.12383 (5)	0.47033 (12)	0.44987 (6)	0.02465 (15)	
H1	0.067688	0.446104	0.408353	0.030*	
C15	0.72309 (5)	0.54097 (11)	0.76693 (6)	0.02409 (15)	
C9	0.37009 (6)	0.20956 (12)	0.46596 (6)	0.02593 (16)	
H9A	0.345701	0.112329	0.486189	0.039*	
H9B	0.432365	0.191579	0.475090	0.039*	
H9C	0.339086	0.228611	0.401702	0.039*	
C2	0.13236 (5)	0.59308 (12)	0.51416 (7)	0.02590 (16)	
H2	0.081895	0.650225	0.516706	0.031*	
C17	0.85370 (5)	0.38883 (12)	0.76148 (5)	0.02227 (14)	
C18	0.83775 (5)	0.14263 (14)	0.66450 (6)	0.02812 (18)	
H18B	0.898124	0.168077	0.665894	0.034*	
H18A	0.802474	0.117384	0.601326	0.034*	
C19	0.83898 (6)	−0.00613 (13)	0.72371 (7)	0.02925 (18)	
H19B	0.865022	−0.099926	0.702348	0.044*	
H19C	0.779012	−0.033514	0.720698	0.044*	
H19A	0.873715	0.019095	0.786277	0.044*	
C20	0.86824 (6)	0.62716 (12)	0.86467 (8)	0.0319 (2)	
H20A	0.839194	0.735725	0.859387	0.038*	
H20B	0.926070	0.643783	0.856798	0.038*	
C21	0.88084 (7)	0.55668 (14)	0.95736 (7)	0.0341 (2)	
H21A	0.909420	0.449030	0.962662	0.051*	
H21B	0.823795	0.544084	0.966166	0.051*	
H21C	0.917496	0.631204	1.003387	0.051*	

### 1,3-Diethyl-2-sulfanylidene-5-[2-(1,3,3-trimethylindolin-2-ylidene)ethylidene]dihydropyrimidine-4,6(1H,5H)-dione (DTB) Atomic displacement parameters (Å^2^)

**Table:**

	*U*^11^	*U*^22^	*U*^33^	*U*^12^	*U*^13^	*U*^23^
S1	0.01270 (8)	0.04879 (16)	0.03404 (12)	−0.00224 (8)	0.00332 (7)	−0.00092 (10)
O1	0.0176 (2)	0.0376 (4)	0.0168 (2)	0.0010 (2)	0.00299 (18)	−0.0047 (2)
N1	0.0150 (2)	0.0200 (3)	0.0174 (2)	−0.0009 (2)	0.00355 (18)	−0.00432 (19)
O2	0.0243 (3)	0.0270 (3)	0.0515 (5)	0.0064 (3)	−0.0069 (3)	−0.0139 (3)
N2	0.0135 (2)	0.0331 (4)	0.0167 (2)	−0.0002 (2)	0.00410 (18)	0.0020 (2)
N3	0.0161 (3)	0.0216 (3)	0.0269 (3)	−0.0032 (2)	−0.0013 (2)	0.0042 (2)
C7	0.0150 (2)	0.0183 (3)	0.0143 (2)	−0.0008 (2)	0.00274 (19)	−0.0018 (2)
C5	0.0142 (2)	0.0190 (3)	0.0167 (2)	−0.0017 (2)	0.00338 (19)	−0.0013 (2)
C8	0.0144 (2)	0.0196 (3)	0.0145 (2)	−0.0008 (2)	0.00288 (19)	−0.0009 (2)
C4	0.0147 (2)	0.0187 (3)	0.0169 (3)	−0.0005 (2)	0.00356 (19)	−0.0012 (2)
C12	0.0141 (3)	0.0240 (3)	0.0176 (3)	0.0006 (2)	0.0031 (2)	0.0000 (2)
C14	0.0143 (3)	0.0221 (3)	0.0186 (3)	−0.0005 (2)	0.0009 (2)	0.0033 (2)
C13	0.0152 (3)	0.0216 (3)	0.0185 (3)	0.0005 (2)	0.0020 (2)	0.0021 (2)
C16	0.0141 (2)	0.0290 (4)	0.0146 (2)	−0.0001 (2)	0.00368 (19)	0.0033 (2)
C10	0.0197 (3)	0.0189 (3)	0.0181 (3)	−0.0024 (2)	0.0035 (2)	−0.0010 (2)
C3	0.0179 (3)	0.0238 (3)	0.0248 (3)	0.0023 (3)	0.0059 (2)	−0.0021 (3)
C6	0.0165 (3)	0.0234 (3)	0.0209 (3)	−0.0043 (2)	0.0016 (2)	−0.0020 (2)
C11	0.0228 (3)	0.0225 (3)	0.0153 (3)	−0.0004 (3)	0.0056 (2)	−0.0004 (2)
C1	0.0149 (3)	0.0276 (4)	0.0277 (4)	−0.0029 (3)	0.0016 (2)	0.0017 (3)
C15	0.0176 (3)	0.0191 (3)	0.0286 (4)	0.0006 (2)	−0.0023 (2)	0.0022 (3)
C9	0.0231 (3)	0.0254 (4)	0.0280 (4)	−0.0003 (3)	0.0065 (3)	−0.0112 (3)
C2	0.0158 (3)	0.0275 (4)	0.0327 (4)	0.0022 (3)	0.0054 (3)	0.0008 (3)
C17	0.0144 (3)	0.0302 (4)	0.0207 (3)	−0.0028 (3)	0.0037 (2)	0.0056 (3)
C18	0.0166 (3)	0.0485 (6)	0.0202 (3)	0.0036 (3)	0.0072 (2)	−0.0065 (3)
C19	0.0206 (3)	0.0327 (4)	0.0337 (4)	0.0031 (3)	0.0078 (3)	−0.0107 (3)
C20	0.0221 (4)	0.0202 (4)	0.0415 (5)	−0.0045 (3)	−0.0061 (3)	−0.0001 (3)
C21	0.0306 (4)	0.0283 (4)	0.0324 (4)	0.0041 (3)	−0.0048 (3)	−0.0082 (3)

### 1,3-Diethyl-2-sulfanylidene-5-[2-(1,3,3-trimethylindolin-2-ylidene)ethylidene]dihydropyrimidine-4,6(1H,5H)-dione (DTB) Geometric parameters (Å, º)

**Table:**

S1—C17	1.6683 (8)	C10—H10B	0.9800
O1—C16	1.2354 (10)	C10—H10C	0.9800
N1—C5	1.4135 (10)	C3—H3	0.9500
N1—C8	1.3462 (9)	C3—C2	1.4024 (12)
N1—C9	1.4585 (11)	C6—H6	0.9500
O2—C15	1.2329 (12)	C6—C1	1.3974 (12)
N2—C16	1.4152 (10)	C11—H11A	0.9800
N2—C17	1.3766 (11)	C11—H11B	0.9800
N2—C18	1.4829 (12)	C11—H11C	0.9800
N3—C15	1.4128 (11)	C1—H1	0.9500
N3—C17	1.3825 (13)	C1—C2	1.3915 (14)
N3—C20	1.4852 (12)	C9—H9A	0.9800
C7—C8	1.5362 (10)	C9—H9B	0.9800
C7—C4	1.5154 (10)	C9—H9C	0.9800
C7—C10	1.5414 (11)	C2—H2	0.9500
C7—C11	1.5460 (10)	C18—H18B	0.9900
C5—C4	1.3907 (10)	C18—H18A	0.9900
C5—C6	1.3871 (10)	C18—C19	1.5194 (16)
C8—C12	1.4027 (10)	C19—H19B	0.9800
C4—C3	1.3850 (11)	C19—H19C	0.9800
C12—H12	0.9500	C19—H19A	0.9800
C12—C13	1.3858 (11)	C20—H20A	0.9900
C14—C13	1.4043 (10)	C20—H20B	0.9900
C14—C16	1.4384 (12)	C20—C21	1.5109 (17)
C14—C15	1.4483 (12)	C21—H21A	0.9800
C13—H13	0.9500	C21—H21B	0.9800
C10—H10A	0.9800	C21—H21C	0.9800
			
C5—N1—C9	122.03 (6)	C7—C11—H11A	109.5
C8—N1—C5	111.58 (6)	C7—C11—H11B	109.5
C8—N1—C9	126.30 (7)	C7—C11—H11C	109.5
C16—N2—C18	115.62 (7)	H11A—C11—H11B	109.5
C17—N2—C16	124.48 (7)	H11A—C11—H11C	109.5
C17—N2—C18	119.75 (7)	H11B—C11—H11C	109.5
C15—N3—C20	115.50 (8)	C6—C1—H1	119.5
C17—N3—C15	124.17 (7)	C2—C1—C6	120.98 (7)
C17—N3—C20	119.97 (7)	C2—C1—H1	119.5
C8—C7—C10	113.77 (6)	O2—C15—N3	119.20 (8)
C8—C7—C11	110.25 (6)	O2—C15—C14	124.33 (8)
C4—C7—C8	101.13 (6)	N3—C15—C14	116.46 (8)
C4—C7—C10	109.95 (6)	N1—C9—H9A	109.5
C4—C7—C11	110.14 (6)	N1—C9—H9B	109.5
C10—C7—C11	111.16 (6)	N1—C9—H9C	109.5
C4—C5—N1	108.74 (6)	H9A—C9—H9B	109.5
C6—C5—N1	128.44 (7)	H9A—C9—H9C	109.5
C6—C5—C4	122.81 (7)	H9B—C9—H9C	109.5
N1—C8—C7	109.09 (6)	C3—C2—H2	119.7
N1—C8—C12	122.01 (7)	C1—C2—C3	120.67 (8)
C12—C8—C7	128.89 (6)	C1—C2—H2	119.7
C5—C4—C7	109.42 (6)	N2—C17—S1	121.36 (7)
C3—C4—C7	131.01 (7)	N2—C17—N3	117.32 (7)
C3—C4—C5	119.57 (7)	N3—C17—S1	121.31 (6)
C8—C12—H12	118.7	N2—C18—H18B	109.5
C13—C12—C8	122.66 (7)	N2—C18—H18A	109.5
C13—C12—H12	118.7	N2—C18—C19	110.68 (7)
C13—C14—C16	123.32 (7)	H18B—C18—H18A	108.1
C13—C14—C15	115.97 (8)	C19—C18—H18B	109.5
C16—C14—C15	120.66 (7)	C19—C18—H18A	109.5
C12—C13—C14	129.01 (8)	C18—C19—H19B	109.5
C12—C13—H13	115.5	C18—C19—H19C	109.5
C14—C13—H13	115.5	C18—C19—H19A	109.5
O1—C16—N2	118.64 (8)	H19B—C19—H19C	109.5
O1—C16—C14	124.72 (7)	H19B—C19—H19A	109.5
N2—C16—C14	116.59 (7)	H19C—C19—H19A	109.5
C7—C10—H10A	109.5	N3—C20—H20A	109.3
C7—C10—H10B	109.5	N3—C20—H20B	109.3
C7—C10—H10C	109.5	N3—C20—C21	111.43 (8)
H10A—C10—H10B	109.5	H20A—C20—H20B	108.0
H10A—C10—H10C	109.5	C21—C20—H20A	109.3
H10B—C10—H10C	109.5	C21—C20—H20B	109.3
C4—C3—H3	120.6	C20—C21—H21A	109.5
C4—C3—C2	118.80 (8)	C20—C21—H21B	109.5
C2—C3—H3	120.6	C20—C21—H21C	109.5
C5—C6—H6	121.4	H21A—C21—H21B	109.5
C5—C6—C1	117.15 (8)	H21A—C21—H21C	109.5
C1—C6—H6	121.4	H21B—C21—H21C	109.5

### 1,3-Diethyl-2-sulfanylidene-5-[2-(1,3,3-trimethylindolin-2-ylidene)ethylidene]dihydropyrimidine-4,6(1H,5H)-dione (DTB) Hydrogen-bond geometry (Å, º)

**Table:**

*D*—H···*A*	*D*—H	H···*A*	*D*···*A*	*D*—H···*A*
C12—H12···O1	0.95	2.28	2.9000 (10)	122
C19—H19*B*···S1^i^	0.98	2.85	3.5573 (9)	130
C21—H21*A*···S1	0.98	2.98	3.4965 (12)	114

Symmetry code: (i) −*x*+2, *y*−1/2, −*z*+3/2.
